# Beyond the Transnosographic Emphasis on Psychosis: Nosological Perspectives on Schizophrenia and Its Prevention

**DOI:** 10.3389/fpsyt.2019.00666

**Published:** 2019-09-18

**Authors:** Anna Comparelli, Andrea Raballo, Maurizio Pompili, Silvana Galderisi

**Affiliations:** ^1^Department of Psychiatry, Sant'Andrea Hospital of Rome, Rome, Italy; ^2^Division of Psychiatry, Clinical Psychology and Rehabilitation, Department of Medicine, University of Perugia, Perugia, Italy; ^3^Department of Neurosciences, Mental Health and Sensory Organs, Suicide Prevention Center, Sant'Andrea Hospital, Sapienza University of Rome, Rome, Italy; ^4^Department of Psychiatry, University of Campania Luigi Vanvitelli, Naples, Italy

**Keywords:** schizophrenia, early psychosis, nosology, extended psychosis phenotype, psychosis spectrum

There is an ongoing debate in the literature regarding relative merits and weaknesses of the concept of transnosographic psychosis, namely the fact that psychosis is not limited to any specific psychotic disorder but rather represents a continuous expression across the spectrum of psychoses and may be perceived as an extreme expression of quantitative traits continuously distributed in the general population. The genesis of this concept is complex and partly derived from research on the early detection of psychosis. Even within the established categorical system, in the last edition of the DSM (1), the chapter “Schizophrenia Spectrum and Other Psychotic Disorders” aimed to capture the underlying dimensional structure of psychosis. At this point in the debate, while scholars discuss a change of name of schizophrenia, an overview of possible nosological perspectives for schizophrenia is a mandatory step, both for clinical practice and for research.

The notion of a broad psychotic spectrum was strongly advocated by Guloksuz and van Os (2), who provocatively announced the “slow death” of the concept of schizophrenia, redefined as the poor outcome fraction of a “truly complex, multidimensional psychotic syndrome.” According to this view, in around 10–20% of the non-ill general population (3), subtle alterations in processing environmental stimuli during daily life give rise to momentary and fluctuating paranoid feelings, or negative affective states (microphenotype). Some of these states tend to persist over time and occur in the presence of other symptoms such as depression and anxiety, giving rise to noticeable experiences of reality distortion (extended phenotype). The copresence of affective dysregulation and reality distortion (psychotic experiences) increases the risk of onset of psychotic disorder (illness macrophenotype), in a sort of psychopathological network of symptoms causally impacting each other over time. In this view, schizophrenia represents a rare mental disorder with poor prognosis within a broader spectrum of psychotic disorders.

The extended psychosis phenotype is trans-nosographic in nature, implying that it is not restricted to any specific psychotic disorder but rather represents both a continuous expression across the psychosis spectrum and an extreme expression of continuously distributed quantitative traits in the general population.

This model should be compared with the paradigm of clinical high risk (CHR), which is conditionally meant to identify those at risk of psychosis among symptomatic, help-seeking subjects.

The CHR paradigm (4–6) was developed with the description of three possible prodromal syndromes—attenuated positive symptom syndrome (APS), genetic risk and deterioration (GRD) and/or brief intermittent psychotic syndrome (BIPS). APS requires the presence of at least one attenuated positive psychotic symptom (unusual thought content, suspiciousness, grandiose ideas, perceptual abnormalities, or disorganized communication) of insufficient severity to meet diagnostic criteria for a psychotic disorder. The attenuated psychotic symptom(s) had to have begun or worsened in the past year. The GRD requires a combination of both functional decline (at least a 30% decrease in Global Assessment of Function score over the last month compared to 12 months ago) and genetic risk; genetic risk refers to having either schizotypal personality disorder or a first-degree relative with a schizophrenia spectrum disorder. The BIPS state requires the presence of any one or more threshold positive psychotic symptoms (unusual thought content, suspiciousness, grandiosity, perceptual abnormalities, and disorganized communication) that are self-limited and too brief (less than one week) to meet diagnostic criteria for a brief psychotic disorder.

The transition rate to psychosis from a CHR state is approximately 20% over 1 year and 36% within three years; among those who convert, about 60% of diagnostic outcomes develop towards the schizophrenia spectrum and the remaining 40% towards mood-related and atypical forms of psychosis (7). Notably, however, only a minority of patients who do not convert will remit (7%), whereas most patients will remain in a comparable state of impairment with continuing levels of attenuated psychotic-like symptoms and functional impairment (30%); others will develop another psychiatric disorder, which is not necessarily associated with psychosis (60%) (8).

Thus, on the one hand the CHR model highlights the existence of a large prevalence of attenuated psychotic symptoms, while on the other it has also been shown to have poor predictive specificity for schizophrenia, since of those who can be classified as CHR, only about 10–15% evolve towards schizophrenia (roughly half of those who convert to full blown psychosis). This is mainly due to two reasons: 1) total exclusion from the CHR criteria of negative and most disorganized symptoms; 2) restriction of the realm of schizophrenia to its mainly delusional–hallucinatory overt forms (9).

Indeed, since the initial conceptualization of CHR status, it was clear that the severity of specific symptoms at baseline (bizarre thinking, social withdrawal, and reduced global functioning) was related to transition to schizophrenia (4, 10, 11). Furthermore, consistent with basic symptom and self-experience disorder research, specific subjective disturbances were found to be predictive of transition to schizophrenia (12).

If the breadth of these symptoms were included in CHR criteria, they would presumably lead to a more accurate prediction of schizophrenia rather than of first psychotic episode in a broad, trans-nosographic sense; nevertheless, positive symptoms are the central pivot of prodromal syndromes and somehow orient the predictive power of the construct towards delusional–hallucinatory outcomes.

In conclusion, the original dual-output model (transition/no transition), due to its constitutive dimensional structure, has been the breeding ground for the trans-nosographic concept of attenuated psychosis. Indeed, attenuated psychosis is best understood as an early subclinical stage, harbouring the potential for pluripotent, transdiagnostic outcome trajectories, including both psychotic and non-psychotic ones.

Intense scientific debate has taken place around these issues. The main point of discrimination is no longer whether CHR status is sufficiently predictive of schizophrenia, but whether it is specifically predictive of psychotic spectrum disorders or simply a risk condition for other non-psychotic psychiatric conditions (13, 14).

The drafting of the DSM-5 was strongly influenced by the pressing downsizing of the category towards a dimensional approach, to the point that schizophrenia is now part of a group of disorders belonging to a spectrum, sharing, at least on a phenotypic level, some of their characteristics. If we embrace a spectrum approach, a legitimate discussion emerges around the benefits of changing the name of schizophrenia, with the specific scope of going further than a mere semantic revision, but maintaining the original nucleus of the knowledge on schizophrenia.

At this point, it is useful to wonder if only the name should be changed, or the concept of schizophrenia itself. Our problem resembles the debate about psychiatric diseaeses as natural entities vs. descriptive diagnostic categories. Regarding this, Kendler (15) stated that “in our current project to study and justify the nature of psychiatric disorders, we should be largely pragmatic but not lose sight of a fundamental commitment, despite the difficulties associated with the reality of psychiatric illness.” *Reality* is here referred to its philosophical meaning of *natural entity*.

In this light, in the redefinition of the name/concept/entity of schizophrenia, three possible perspectives may be outlined:

1) Schizophrenia as a stochastic result of pathogenetic paths based on persistent interplay of genes and the environment, and framed in the context of the transnosographic psychopathological network, which leads to poor outcomes of clinical dimensions (affective, positive, negative, and cognitive) that are overlapping and severely expressed. In this perspective, schizophrenia would basically denote the most extreme (and somehow residual) outcomes of severe mental illnesses.
2) Schizophrenia as the delusional–hallucinatory expression of the psychotic spectrum that, regardless of pathogenetic and outcome paths, is characterized by the expression of positive symptoms. Such a notion, which is a variant of *einheitpsycose-unitary psychosis*, and includes affective and non-affective clinical dimensions, resembles the more recent DSM orientation and may be extended to schizoaffective disorders as well as to to certain forms of mania.
3) Schizophrenia as a disease tending virtually to Jaspers’ morbid entity, with its etiopathogenesis strongly linked to neurodevelopmental disorders akin to Kahlbaum-Hecker Hebephrenia, as recently overviewed (16). Predominant disorganized/cognitive and negative symptoms would characterize the illness early, even in its prodromal stages, which ought to be detected before, and regardless of the absence of frank-full blown positive psychotic symptoms, in order to avoid natural progression of the disease.

From the perspective of early detection and prevention, in the first case prevention of schizophrenia would imply a general model of universal prevention of severe psychopathology; in the second hypothesis, schizophrenia, being only one of the many disorders that can cause positive symptoms, is detectable through the current concept of CHR based on positive symptoms; in the third scenario, we might think of reformulating a prodromal model by referring to criteria including both cognitive/disorganized, blunted affect and social/academy withdrawal symptoms, and intermediate phenotypes (such as impaired social cognition and speed of processing). While the first two options might seem the smoothest and less problematic ones, the last option might be the most promising in terms of improving scientific knowledge and offering the opportunity of better encompassing effective strategies for early identification.

## Author Contributions

All authors listed have made substantial, direct, and intellectual contribution to the work and approved it for publication.

## Conflict of Interest Statement

The authors declare that the research was conducted in the absence of any commercial or financial relationships that could be construed as a potential conflict of interest.
